# Procyanidin B3 alleviates intervertebral disc degeneration via interaction with the TLR4/MD‐2 complex

**DOI:** 10.1111/jcmm.15074

**Published:** 2020-02-18

**Authors:** Ping Shang, Qian Tang, Zhichao Hu, Shiyuan Huang, Yuezheng Hu, Jianhong Zhu, Haixiao Liu

**Affiliations:** ^1^ Department of Rehabilitation The Second Affiliated Hospital and Yuying Children's Hospital of Wenzhou Medical University Wenzhou China; ^2^ Department of Orthopedic Surgery Shanghai Jiao Tong University Affiliated Shanghai Sixth People’s Hospital Shanghai China; ^3^ Department of Orthopaedic Surgery The Second Affiliated Hospital and Yuying Children's Hospital of Wenzhou Medical University Wenzhou China; ^4^ Department of Preventive Medicine Wenzhou Medical University Wenzhou China

**Keywords:** intervertebral disc degeneration, nucleus pulposus cell, Procyanidin B3

## Abstract

As a chronic musculoskeletal degeneration disease, intervertebral disc degeneration (IVDD) has been identified as a crucial cause for low back pain. This condition has a prevalence of 80% among adults without effective preventative therapy. Procyanidin B3 (Pro‐B3) is a procyanidin dimer, which is widely present in the human diet and has multiple functions, such as preventing inflammation. But the inhibiting effect of Pro‐B3 in IVDD development is still no known. Thus, our study aimed to demonstrate the therapeutical effect of Pro‐B3 in IVDD and explain the underlying mechanism. In vitro studies, human nucleus pulposus (NP) cells were isolated and exposed in lipopolysaccharide (LPS) to simulate IVDD development. Pro‐B3 pre‐treatment inhibited LPS‐induced production of inflammation correlated factors such as tumour necrosis factor α (TNF‐α), interleukin‐6 (IL‐6), prostaglandin E2 (PGE2) and Nitric oxide (NO). On the other hand, LPS‐medicated extracellular matrix (ECM) breakdown was blocked in Pro‐B3 treated NP cells. Additionally, Pro‐B3 treatment blocked the activation of NF‐κB/toll‐like receptor 4 pathway in LPS‐exposed NP cells. Mechanistically, Pro‐B3 could occupy MD‐2's hydrophobic pocket exhibiting high affinity for LPS to intervene LPS/TLR4/MD‐2 complex formation. In vivo, Pro‐B3 treatment prevented the loss of gelatin NP cells and structural damage of annulus fibrosus in rat IVDD model. In brief, Pro‐B3 is considered to be a treatment agent for IVDD.

## 1 INTRODUCTION

Intervertebral disc degeneration (IVDD) is recognized as the leading contributor to the low back pain, affecting about 80% of adults and surges global medical spending.^1^ Clinical treatments for IVDD or low back pain are focus on the relieving of symptoms while little controlling over the IVDD development. Currently, there are several risk factors are associated with IVDD initiation and development such as ageing, sexuality and obesity, but the pathogenesis and aetiology of IVDD are not sufficiently clear.^2^ As the avascular organ, three cell types exist in intervertebral disc, including the endplate chondrocytes (EP), inner and outer annulus fibrosus (AF) cells, and the internal nucleus pulposus (NP) cells.^3^ NP cells could produce extracellular matrix (ECM) molecules to stabilize the biomechanical equilibrium and structure of intervertebral disc.^4^

During IVDD process, multiple inflammatory cytokines and catabolic factors accumulate in NP tissue, subsequently affects the viability and function of NP cells. Accumulating evidence demonstrated that lipopolysaccharide (LPS) induced inflammatory cytokines production and ECM degeneration in 2D or 3D cultured NP cells.^5^, ^6^, ^7^ In addition, the level of LPS and polysaccharide‐binding protein (LBP) increases in serum of patients with IVDD relative to the unaffected individuals.^8^ Therefore, we perform LPS as a stimulating factor in our in vitro experiments of IVDD.

As a transmembrane protein in mammals, toll‐like receptor‐4 (TLR4) regulates various inflammatory signings such as NF‐κB pathway. Myeloid differentiation protein‐2 (MD‐2) recognizes LPS to create TLR4/MD‐2/LPS complex, subsequently recruit tumour necrosis factor receptor‐related factor 6 (TRAF6), myeloid differentiation factor 88 (MyD88) and interleukin‐1 receptor‐related kinases (IRAKs) to initiate the nuclear transfer of p65.^9^, ^10^ During the NF‐κB pathway activation, p65 transfers to nuclear and interacts with promoter region of genes to induce the inflammatory and catabolism factors production.^11^, ^12^ The level of TRAF6, MyD88 and TLR4 proteins is increased and NF‐κB pathway is activated in IVDD tissue, compared to the healthy human beings.^8^, ^13^ TLR4/NF‐κB could be considered as the promising therapeutic target for IVDD.

Procyanidins belong to the nature nutraceuticals abundant in many plants such as grape, cocoa and seeds.^14^ Procyanidin B3 (Pro‐B3) is a procyanidin B‐type dimer and possess multiple activities, for example anti‐cancer, anti‐oxidative stress and anti‐inflammation.^14^, ^15^, ^16^ It is worth mention that the preventive effect of Pro‐B3 on cartilage degradation has been proved by surgically induced osteoarthritis model.^17^ For mechanism, Pro‐B3 could prevent the chondrocyte apoptosis under oxidative condition. Besides, treating the primary human OA chondrocytes with Procyanidin complex including Pro‐B2 and Pro‐B3 individually reduces level of ADAMTS5, MMPs, IL‐6, IL‐1β and TNF‐α^18^ Given the evidence discussed above, we postulated that Pro‐B3 may delay IVDD progression. Thus, we exposed NP cells to LPS and created a rat IVDD model to assess the positive effects of Pro‐B3 and the mechanisms involved.

## 2 EXPERIMENTAL PROCEDURES

### 2.1 Reagents

Procyanidin B3 (purity 95%) was obtained from BOC sciences. Other reagents used were as follows: primary antibodies for p65, IκBα and COX‐2 (CST), GADPH, TRAF6, MyD88, ADAMTS5, Lamin B, collagen Ⅱ and TLR4 (Abcam). Anti‐IRAK1, goat antimouse and goat anti‐rabbit IgG‐HRP were bought from Bioworld. Recombined human TLR4 and MD2 protein (rhTLR4 and rhMD2) were bought from R&D Systems. Anti‐MD‐2 and Biotin‐labelled LPS (Biotin‐LPS) were obtained from eBioscience. Type II collagenase, fast green and Safranin‐O were bought from (Sigma‐Aldrich) and Alexa Fluor^®^488 labelled Goat Anti‐Rabbit IgG (H + L) secondary antibody (Jackson ImmunoResearch).

### 2.2 Primary human NP cultures

The collection of human NP tissues was sanctioned by the Medical Ethical Committee of the Second Affiliated Hospital in line with the regulations of the Declaration of Helsinki. Human intervertebral disc (IVD) specimen was harvested from five lumbar vertebral fracture patients (aged 20‐32 years, two women and three men) who suffer posterior discectomy, spinal fusion, decompression and stability at our unit. All patients provide an agreement to provide specimen. The gel‐like NP specimen was isolated and washed thrice with PBS. Thereafter, tissue incubated with 0.2% type II collagenase and 0.25% trypsin (Gibco), for digestion, for 3 hours at 37°C. NP cells were incubated in flasks with DMEM/F12 comprised 10% FBS and antibiotic. At about 80% confluence, NP cells were cultured without serum overnight and replanted into new culture. The 2nd passage NP cells were used.

### 2.3 Animal model

Thirty mature male Sprague‐Dawley rats (220‐250 g) were utilized and all of the experimental procedures were in line with the Guide for the Care and Use of Laboratory Animals of the National Institutes of Health and got alienation of the Animal Use and Care Committee of our unit. We categorized all animals to three groups: IVDD + Pro‐B3 group, IVDD group and Control group. IVDD model was created as detailed previously.^19^ In a phrase, 2% (w/v) pentobarbital (40 mg/kg) was given to the animals by intraperitoneal injection. The digital palpation method was used to locate the tail disc (Co7/8) on the coccygeal vertebrae, followed by confirmation by trial radiograph. The entire layer of annulus fibrosus (AF) was punctured via tail‐skin with needles (21G). During the puncturing procedure, a 5 mm length needle was used to avoid deep punctures. This length was preliminarily tested based on the NP and AF dimensions. Each needle was kept within the disc for 1 minute and rotated 360°. The investigators who performed the surgery were not aware of the animal groups. All animals were daily monitored and allowed freedom to bear weight and engage in activity.

### 2.4 Experimental design

To explore the protective ability of Pro‐B3 in vitro, the NP cells were exposed to 1 µg/mL LPS, singly or together with Pro‐B3 pre‐treatment at various doses (2.5, 10, 40 µmol/L). In the control group, the treatment was omitted but the media were changed. To unravel the mechanism of Pro‐B3 protection (40 µmol/L), the duration and dose of LPS were 2 hours and 1 µg/mL. To assess the functional changes, for example markers of inflammation and ECM, the duration of LPS was prolonged to 24 hours.

For in vivo experiments, IVDD + Pro‐B3 group was administered Pro‐B3 dissolved in water (30 mg/kg/d) by oral gavage for 4 weeks after surgery. On the other hand, rats of IVDD group were administered an equivalent amount of water. Experimental rats were killed at 1 month after operation and collected rat tail disc (Co7/8) tissue sample for histological examination.

### 2.5 Cell viability determination

The 2nd passage NP cells (8000 cells/well of 96‐well plate) were treated with Pro‐B3 (0, 1, 2.5, 10, 40, 160 µmol/L) for 24 hours in a medium without serum. CCK‐8 kit was used to measure cell viability as described in producer's protocol (Dojindo Co). All tests were repeated 5 times.

### 2.6 NO levels and ELISA

Griess reagent was applied in the determination of interaction of NO in the medium. After the treatment of NP cells, culture media were used to quantify ADAMTS‐5, MMP13, aggrecan, collagen II, IL‐6, TNF‐α and PGE levels in the supernatant by ELISA kits. All tests were repeated 5 times.

### 2.7 Western blotting

After treatments, NP cells were prepared by washing with cold PBS followed by lysing with cooled RIPA lysis solution plus 1 mmol/L PMSF and followed by lysate and centrifugation. The concentrations of each protein were detected by BCA kit (Beyotime). Equal protein lysates (40 ng) were resolved SDS PAGE and then transferred to PVDF membrane (Bio‐Rad). This was followed by a blocking‐step using 5% fat‐free milk for 2 hours, and incubation with primary antibodies for IκBα (1:1000), p65 (1:1000), COX‐2 (1:1000), iNOS (1:1000), GADPH (1:5000), and Lamin B1 (1:1000), TLR4 (1:500), MyD88 (1:1000), IRAK1 (1:500) and MD‐2 (1:300), diluted in 2% TBST. Membrane was then dipped in 2nd antibody solution for 2 hours, followed a wash‐out step with TBST 3 times. Finally, the blots were imaged with electrochemiluminescence plus reagent (Invitrogen) and quantification was done by Image Lab 3.0 software (Bio‐Rad).

### 2.8 Immunofluorescence

NP cells in 6‐well glass plates were incubated with LPS or co‐incubated with 1 µg/mL LPS and 40 µm Pro‐B3 for 24 hours after which they were stained for ADAMTS‐5 and collagen II. To perform p65 staining, LPS treatment period was shortened to 2 hours. After treatment and washing with 500 µL of 1× PBS, they were fixed using paraformaldehyde and then permeated by 400 µL of 0.1% Triton X‐100 in 1× PBS solution for 15 minutes. Thereafter, add 5% BSA at 37°C for 1 hour as a blocker for non‐specific binding. Subsequent to this, PBS was used to wash the cells followed by incubation with primary antibodies: ADAMTS5 (1:100), collagen Ⅱ (1:100) and p65 (1:100) in a humidified incubator for 24 hours at 4°C. After treatments, cells were washed and treated with Alexa Fluor^®^488 labelled conjugated 2nd antibody (1:200) for 1 hour at room temperature and then labelled with DAPI (Invitrogen) for 5 minutes. Fluorescence microscopy was applied to examine the cells.

The immunofluorescence staining was also performed in a vivo study. Following dehydrated and embedded in paraffin, the tissues were cut into 5‐µm sagittal sections. For immunofluorescence, sections were deparaffinized in xylene and rehydrated by ethanol washes. And, sections were incubated with 10% bovine serum albumin for 1 hour at room temperature in PBS containing Triton X‐100. They were then incubated with primary antibodies overnight at 4°C in the PBST. After primary antibody incubation, sections were washed for four times for 10 minutes at room temperature and then incubated with Alexa Fluor 488 Goat anti‐rabbit secondary antibody for 1 hour at room temperature. Sections were rinsed three times with PBS and incubated with 4,6‐diamidino‐2‐phenylindole (DAPI) for 10 minutes and finally washed in PBS and sealed with a coverslip. The images were captured with a fluorescence microscope (Olympus Inc, Tokyo, Japan), and the rate of MD‐2 positive cells each section was quantitated by observers who were blinded to the experimental groups.

### 2.9 Molecular modelling

The ChemBio3D and ChemBioDraw tools were used to draw the molecular structure of Pro‐B3 while minimizing energy. The human MD2/lipid IVa complex (PDB code 2E59) was retrieved from PDB (https://www.rcsb.org/), then subjected to docking. PyMoL (version 1.7.6) was used to minimize and default parameters were used for the lowest energy conformations. The protein‐ligand docking analysis provided binding pocket moieties binding flexibility with ligand by using AutoDockTools (version 1.5.6). UCSF PyMoL was used for final image generation.

### 2.10 MD‐2 and TLR4 competitive ELISA test

To assess the capability of Pro‐B3 compromising the binding between LPS and MD‐2/TLR4, we performed a cell‐free competitive ELISA. Human anti‐TLR4 or anti‐MD‐2 were attached to walls of 96‐well plate using 10 mmol/L Tris‐HCl buffer (pH 7.5). After incubation, the plates washed with poly (butylene succinate‐co‐butylene terephthalate) (PBST). Following blocking, recombinant toll‐like receptor 4 (rhTLR4) or recombinant human MD‐2 (rhMD‐2) was mixed with 10 mmol/L Tris‐HCl buffer (pH 7.5) followed by incubation and washing of the plates with PBST. Next, biotin‐LPS containing Pro‐B3 (0, 2.5, 10 or 40 µmol/L) was added. This was followed by addition of streptavidin‐conjugated horseradish peroxidase (Beyotime) 1 hour at room temperature. The SpectraMax M5 plate reader (Molecular Devices) was used to determine the activity of Horseradish peroxidase at 450 nm, in the presence of tetramethylbenzidine substrate.

### 2.11 Immunoprecipitation

After treatments, cell lysates were prepared and then incubated with anti‐TLR4 for 1 hour. The protein G‐Sepharose beads were used to retrieve the immune complexes at 4°C overnight. We washed the precipitates thrice with ice‐cold PBS. The samples were boiled with reagent buffer and the level of MD‐2 was measured by immunoblotting with anti‐MD‐2 (IB).

### 2.12 Histopathologic analysis

The animals were killed at 4 weeks post‐puncture. The tail disc specimen (Co7/8) was obtained. After fixation, it was decalcified and embedded in paraffin and serially sectioned. HE and S‐O staining was performed and then three sections from each specimen were captured under a microscope. The morphology and cellularity of AF and NP were assessed with grading scale by investigators unaware of the groupings. Histologic scores were as follows: normal disc (5), moderate degeneration of disc (6‐11) and severe degeneration of disc (12‐15).^19^

### 2.13 Magnetic resonance imagings (MRI) assay

The rats were divided into 3 groups: control, IVDD and IVDD + Pro‐B3 group. MRI assay was performed at 4 weeks for rat after puncture. MRI was performed to evaluate the signal and structural changes in sagittal T2‐weighted images using a 3.0 T clinical magnet (Philips Intera Achieva 3.0MR). T2‐weighted sections in the sagittal plane were obtained in the following settings: fast spin echo sequence with time to repetition (TR) of 5400 ms and time to echo (TE) of 920 ms; 320 (h) 9256 (v) matrix; field of view of 260; and 4 excitations. The section thickness was 2 mm with a 0‐mm gap. The MRIs were evaluated by another blinded orthopaedic researcher using the classification of intervertebral disc degeneration as reported by Pfirrmann et al^20^ (1 point = Grade I, 2 points = Grade II, 3 points = Grade III, 4 points = Grade IV, 5 points = Grade V).

### 2.14 Data analysis

The tests were repeated 5 times. One‐way analysis of variance followed by the Tukey's test was to compare groups. Values are shown as mean ± SD SPSS 20.0 were applied for statistical analyses. Non‐parametric data (like histopathologic score) were assessed by the Kruskal‐Wallis *H* test. *P* values < .05 were considered significant.

## 3 RESULT

### 3.1 The potential cytotoxicity of Procyanidin B3 in nucleus pulposus cells

**Figure 1:**
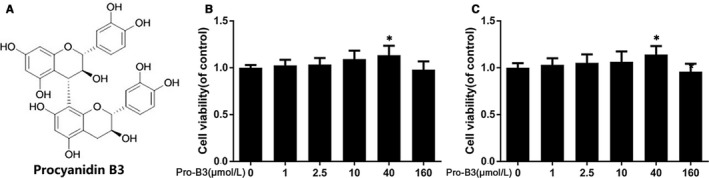
Effect of Pro‐B3 on human NP cells viability. A, Chemical structure of Procyanidin B3. B and C, Cytotoxicity of Pro‐B3 on NP cells at 24 and 48 h. The data in the figures represent the averages ± SD Significant differences among different groups are indicated as *P < .05 vs control group, n = 5

The chemical structure of Pro‐B3 is presented in Figure 1A. We performed the CCK8 test to assess the Pro‐B3 cytotoxicity in NP cells. Figure 1B and C reveal that cells were treated by Pro‐B3 with increasing dose of 0, 1, 2.5, 10, 40 and 160 µmol/L for 24 and 48 hours and results indicated that the Pro‐B3 significantly up‐regulated cell viability with 40 µmol/L for 24 and 48 hours. Thus, we chose the 2.5, 10 and 40 µmol/L Pro‐B3 for subsequent in vitro experiments.

### 3.2 Anti‐inflammatory role of Procyanidin B3 in LPS‐exposed nucleus pulposus cells

**Figure 2:**
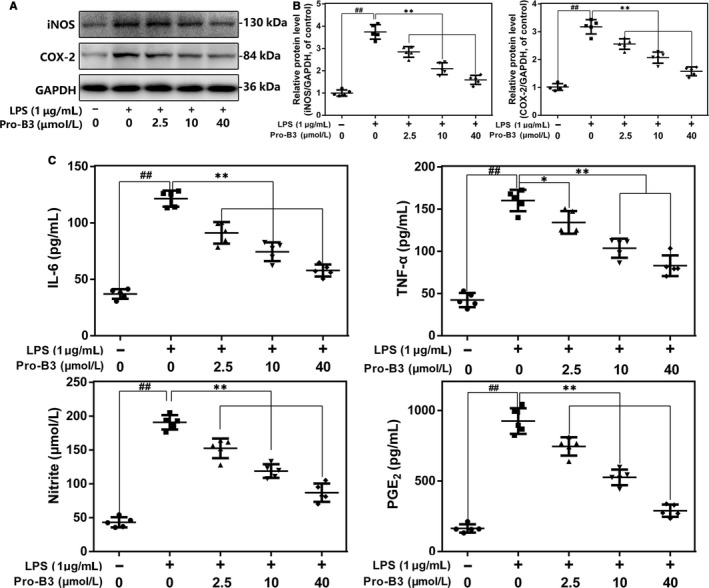
Influence of Pro‐B3 in LPS‐induced inflammatory reaction in human NP cells. A and B, iNOS and COX‐2 protein level in NP cells measured by Western blot. C, Effect of Pro‐B3 on LPS‐exposed IL‐6, TNF‐α, PGE2, and NO production in human NP cells. The data in the figures represent the averages ± SD Significant differences among different groups are indicated as ^##^ *P* < .01, vs control group; **P < .01 vs LPS alone treatment group, n = 5

Inflammation plays an important role in IVDD development. Here, we carried out WB and ELISA to measure the associated inflammatory cytokines production. Results from Western blot showed Pro‐B3 inhibited LPS‐induced increased COX‐2 and iNOS expression (Figure 2A and B). Moreover, the endogenous TNF‐α, PGE2, NO and IL‐6 secretion in NP cells were increased after LPS stimulation, but Pro‐B3 administration reversed them in concentration‐dependent manner by ELISA test (Figure 2C). These data proposed that Pro‐B3 could suppress LPS‐medicated inflammation.

### 3.3 Effect of Procyanidin B3 on ECM degradation and synthesis in LPS‐stimulated nucleus pulposus cells

**Figure 3:**
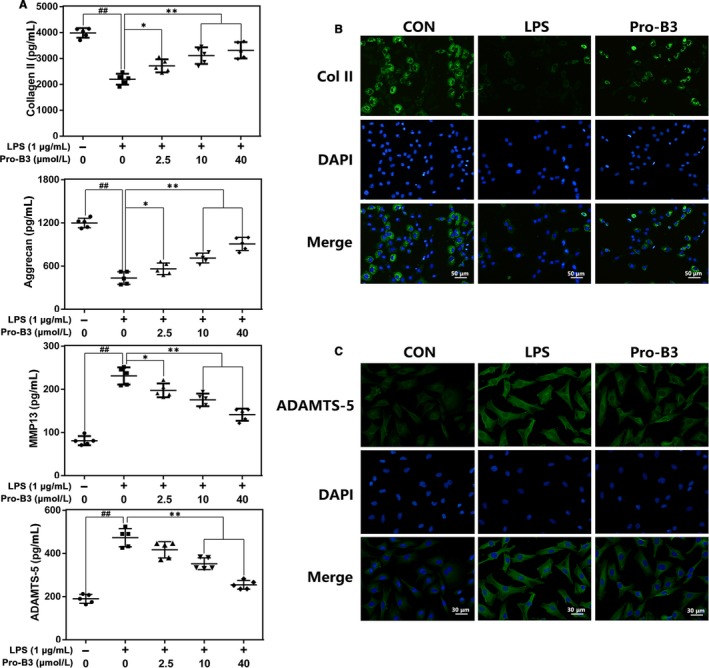
Influence of Pro‐B3 in LPS‐medicated ECM degeneration in human NP cells. A, The level of Collagen II, aggrecan, MMP13 and ADAMTS‐5 in NP cells treated as above were visualized by ELISA. B and C, The representative collagen II and ADAMTS‐5 were detected by the immunofluorescence combined with DAPI staining for nuclei (scale bar: 50 or 30 µm). The data in the figures represent the averages ± SD Significant differences among different groups are indicated as ^##^ *P* < .01, vs control group; *P < .05 and **P < .01 vs LPS alone treatment group, n = 5

The balance of ECM synthesis and degradation contributes the biomechanical equilibrium and structural stability of the intervertebral disc, which is also considered as a key index to estimate the NP cell function. The ELISA provided in Figure 3A found that the collagen II and aggrecan level in LPS‐treated NP cells were down‐regulated, but ADAMTS‐5 and MMP13 were up‐regulated. Interestingly, Pro‐B3 treatment could reverse these trends in concentration‐dependent manner. And immunofluorescence staining data are consistent with ELISA result (Figure 3B and C).

### 3.4 Effect of Procyanidin B3 on NF‐κB pathways activity in LPS‐triggered nucleus pulposus cells

**Figure 4:**
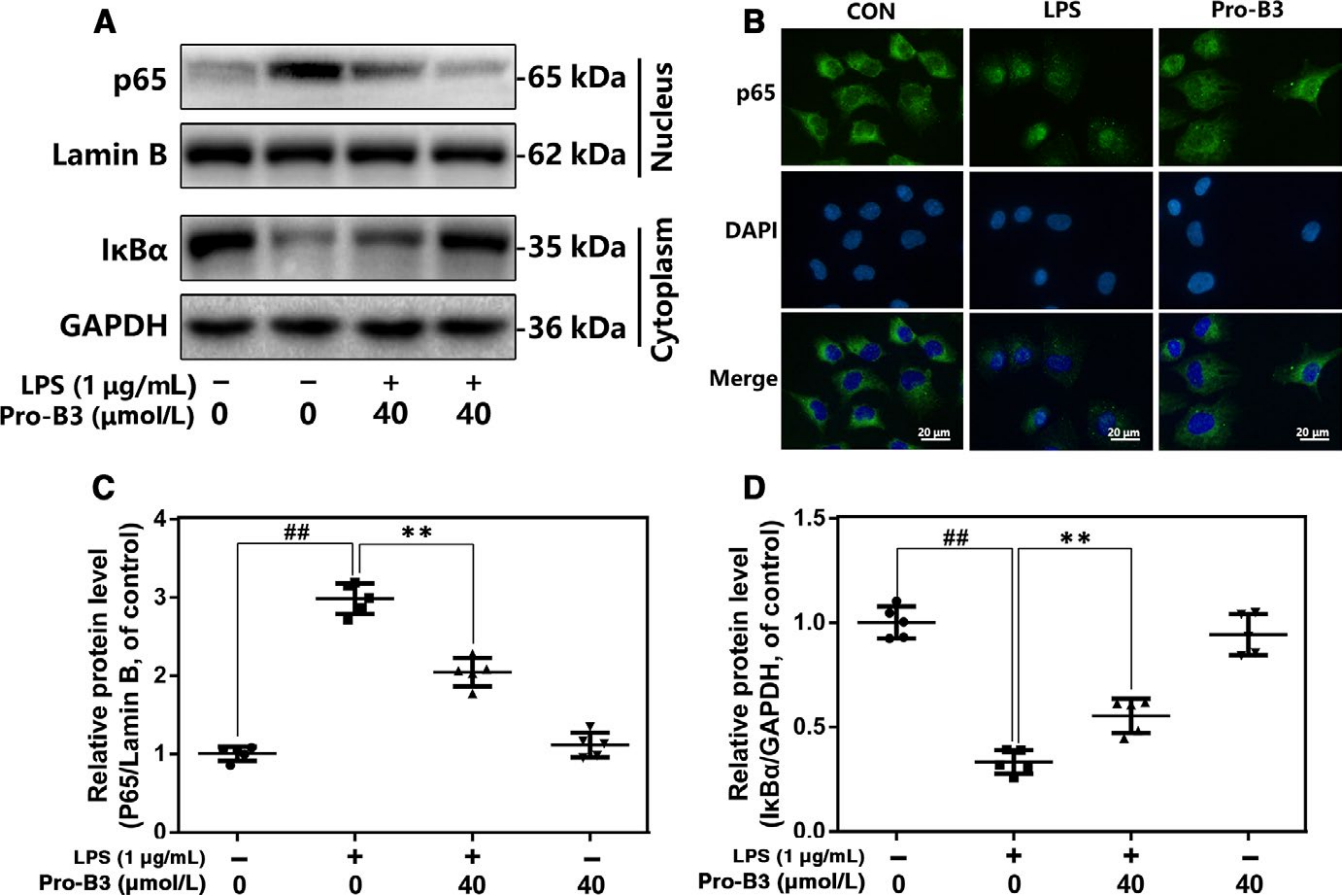
Influence of Pro‐B3 in LPS‐medicated activation of NF‐κB pathway in human NP cells. A, C and D, The protein expressions of IκBα in cytoplasm and p65 in nuclear in NP cells treated as above were detected by Western blot. B, The nuclei translocation of p65 was detected by the immunofluorescence combined with DAPI staining for nuclei (scale bar: 20 µm). The data in the figures represent the averages ± SD Significant differences among different groups are indicated as ^##^ *P* < .01, vs control group; **P < .01 vs LPS alone treatment group, n = 5

To explore the protection mechanism of Pro‐B3 in LPS‐exposed NP cells, we detect the NF‐κB pathway activity by Western blot. Figure 4A, C and D demonstrate that LPS decreased the cytoplasmic IκBα expression but increased nuclear p65 expression, whereas Pro‐B3 treatment reversed these changes. However, Pro‐B3 treatment alone did not affect the activity of NF‐κB signalling. In addition, immunofluorescent staining showed that Pro‐B3 inhibits LPS‐induced p65 nuclear translocation (Figure 4B).

### 3.5 Impact of Procyanidin B3 on TLR4 pathways in LPS‐medicated nucleus pulposus cells

**Figure 5:**
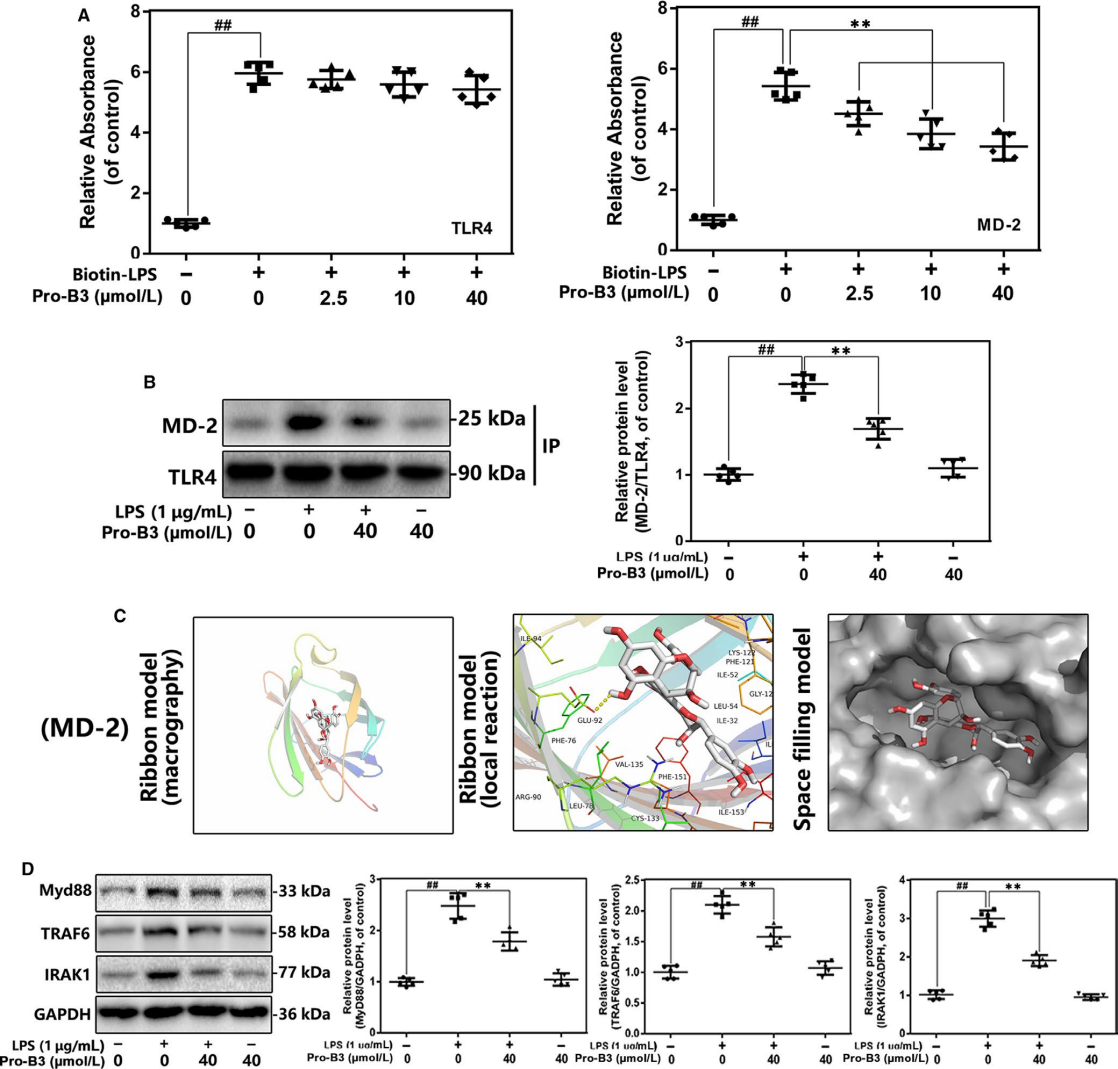
Influence of Pro‐B3 on LPS‐induced TLR4/MD‐2 signalling activation. A, The binding of biotin‐labelled LPS to rhMD‐2 and rhTLR4 was examined by competitive ELISA. B,The complexes of TLR4‐MD‐2 in NP cells treated as above were detected by immunoprecipitation. C, Pro‐B3 was docked with the MD‐2 structure. Docking studies were performed as described in Materials and methods. The protein residues are shown in a ribbon model. The proposed binding pose of Pro‐B3 shows interactions with GLU‐92. The space filling models show the binding of Pro‐B3 in the inhibitory binding pockets. D, The protein expressions of MyD88, IRAK‐1 and TRAF‐6 in NP cells treated as above were detected by Western blot. The data in the figures represent the averages ± SD Significant differences among different groups are indicated as ^##^ *P* < .01, vs control group; **P < .01 vs LPS alone treatment group, n = 5

As the co‐receptor of TLR4, MD‐2 could recognize lipid side chains of LPS by its large hydrophobic pocket. The interaction between LPS and MD‐2 links two TLR4 molecules thereby forming TLR4/MD‐2/LPS complex, which recruits several intracellular adaptor proteins to activate to some downstream signalling including NF‐κB pathway. To investigate the effect of Pro‐B3 in LPS‐activated TLR4 signalling, we performed the following experiments: co‐immunoprecipitation, competitive ELISA and docking analysis. In ELISA analysis (Figure 5A), Pro‐B3 suppressed the interaction of biotin‐LPS with rhMD‐2, whereas does not affect the binding of biotin‐LPS and rhTLR4; suggesting that the inhibitional effect of Pro‐B3 in TLR4/MD‐2/LPS complex formation by intervening the binding of LPS to MD‐2, but not to TLR4. Results in Figure 5B and C showed that Pro‐B3 inhibited the LPS‐mediated crosstalk of MD‐2 and TLR4, but Pro‐B3 treatment alone was not capable of binding of MD‐2 and TLR4, which indicated Pro‐B3 might competitively occupy the binding site to decrease TLR4/MD‐2/LPS complex formation. Additionally, molecular docking assay found the direct affinity of Pro‐B3 and MD‐2 (−7.4 kcal/mol). In terms of molecular structure, Pro‐B3 was fully wrapped within the inhibitory pocket of MD‐2 as revealed by space filling model. And there is a hydrogen bond linking the Pro‐B3 molecule and the GLU‐92 of MD‐2 in ribbon model. These data indicate Pro‐B3 might binds with MD‐2 to suppress the LPS‐stimulated TLR4 signalling.

### 3.6 Influence of Procyanidin B3 on the expression of IRAK‐1, MyD88 and TRAF‐6 in LPS‐activated nucleus pulposus cells

Next, we used Western blot to further the effect of Pro‐B3 in several intracellular adaptors in TLR4 signalling. In the Figure 5D, LPS exposure improves the level expression of MyD88, TRAF‐6 and IRAK‐1, but Pro‐B3 administration significantly suppresses these trends. This result indicated that Pro‐B3‐induced anti‐inflammation protection might be medicated by the prevention of TLR4/MyD88 signalling.

### 3.7 Procyanidin B3 ameliorated IVDD progression in puncture‐induced rat IVDD model

**Figure 6:**
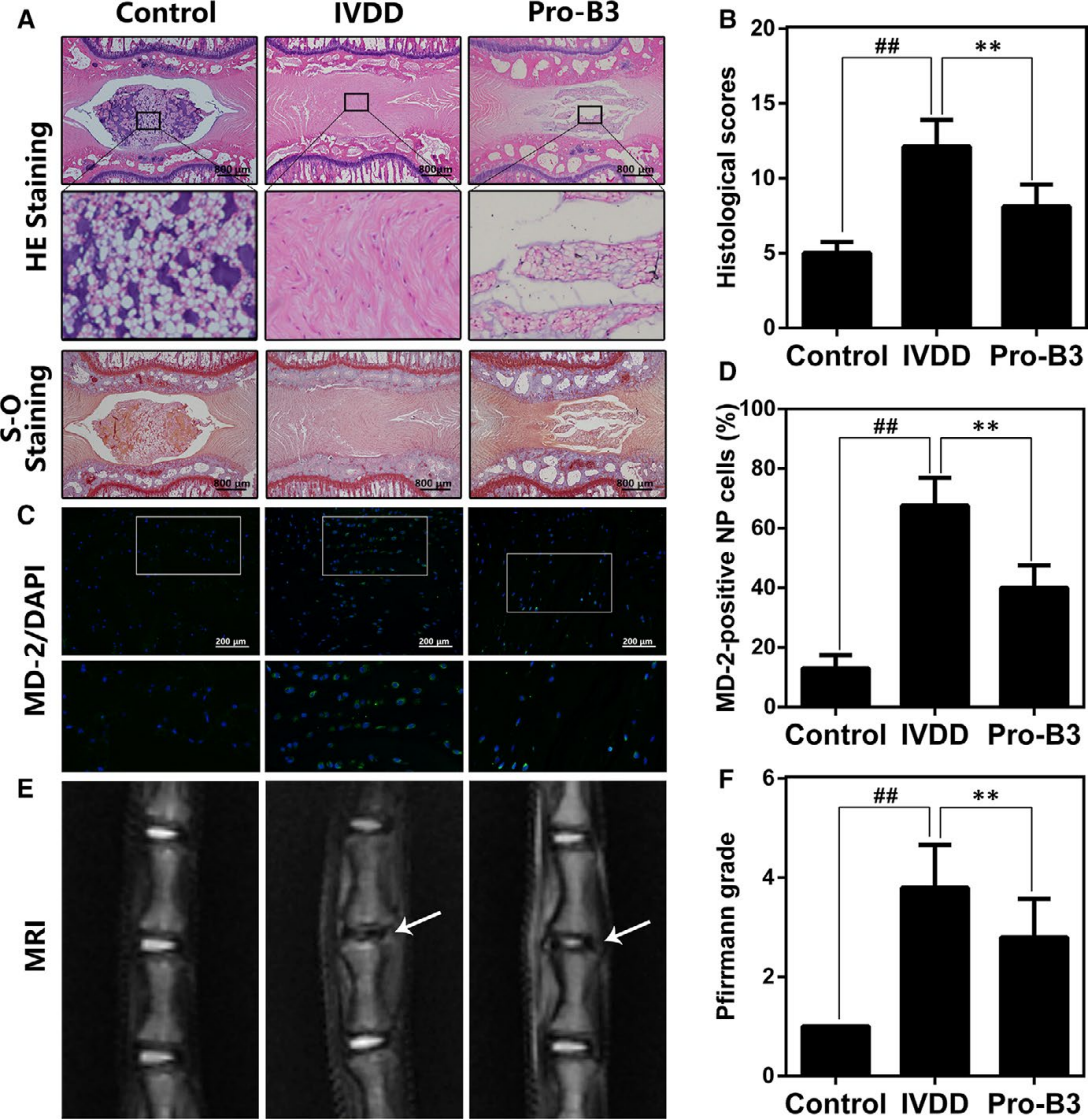
Pro‐B3 attenuates IVDD development in vivo. A, Representative HE and S‐O staining of NP tissue from three groups at 4 wks post‐surgery (scale bar: 800 µm). B, The histological grades evaluated at week 4 in three groups. The data in the figures represent the averages ± SD (C) Representative immunofluorescence staining of MD‐2 from three groups at 4 wks post‐surgery (scale bar: 200 µm). D, Quantitation of immunofluorescence staining of MD‐2 positive cells. E, T2 weighted MRI of a rat tail disc at 4 wks after disc puncture surgery (white arrows). F, The respective Pfirrmann grade scores at 4 wks after disc puncture surgery. Significant differences among different groups are indicated as ^##^ *P* < .01, **P < .01, n = 10

To assess the protection of Pro‐B3 in vivo, we established the puncture‐induced rat IVDD model. HE and S‐O staining and MRI were used to evaluate histomorphology and imageology change for rat intervertebral disc tissue. As shown in Figure 6A, the number of gelatin NP cells decreased and which were substituted by fibrochondrocytes. Structurally, AF displayed a tear or serpentine pattern before inward protrusion in IVDD group. However, Pro‐B3 administration obviously delayed these histopathological changes with less unordered AF and more NP cells. Histological scores also showed that Pro‐B3 groups have a lower score relative to IVDD group (Figure 6B). From MRI results, compared to intervertebral disc in control group, T2‐weighted signal intensity of the intervertebral disc in damaged section was gradually weakened at 4 weeks after puncture, and the degeneration was confirmed by Pfirrmann grade scoring. Interestingly, Pro‐B3 treatement reduced MRI signal intensity weakening and showed higher Pfirrmann grade scores (Figure 6E and F). Futhermore, the number of MD‐2‐positive cells was increased in the IVDD group, while Pro‐B3 treatment decreased the aberrant expression of MD‐2. These results demonstrate that Pro‐B3 delay IVDD development in rats and the mechanism underlying the protection of Pro‐B3 might involve its targeting of MD‐2.

## 4 DISCUSSION

IVDD is a musculoskeletal degeneration disease clinically characterized by prolonged low back pain, and lumbar dysfunction and has not satisfactory treatment.^21^, ^22^ Non‐steroidal anti‐inflammatory drugs (NSAIDs) are the mainstream drugs for relieving IVDD symptoms but not with efficacy for prevention the exacerbation of IVDD. Moreover, NSAIDs produce some adverse drug effects which may carry some risk such as cardiac diseases or stroke, at high doses.^23^ Studies are therefore needed to develop more effective and safer drugs for IVDD treatment.

Procyanidins derive from a variety of plants especially in grape seeds as a complicated mixture of structurally related ingredients including catechin, epicatechin, procyanidins B1‐B5 and procyanidin C1.^24^, ^25^ Pro‐B3 is a catechin dimer and extensively researched due to its abundance in the human diet.^26^ Because of its smaller molecular weight, the gastrointestinal system could absorb it well relative to the procyanidins polymers.^24^ Pervious study reported Pro‐B3 exhibits many bioactive effects such as anti‐inflammation, which has been shown in mouse surgical osteoarthritis model.^17^, ^27^, ^28^ But its protection and specific mechanisms of IVDD disease are not known. Therefore, we attempted to uncover the role of Pro‐B3‐in preventing inflammation in IVDD. Results indicated that Pro‐B3 inhibits LPS‐triggered COX‐2‐PGE2 and iNOS‐NO production and inflammation factors expression by blocking TLR4/NF‐κB pathway. In addition, Pro‐B3 reduced aggrecan and collagen II degeneration as well as ADAMTS‐5 and MMP‐13 expression following LPS exposure. Animal studies showed that Pro‐B3 prevented the progression of IVDD.

In IVDD process, inflammation at local disc tissues is the leading cause. It is reported that multiple inflammatory cytokines such as IL‐6, TNF‐α and IL‐1β were elevated in IVDD patients, but the upstream regulation of them has not been fully explained.^8^ Pan and his colleagues found that serum level of LPS and LBP are strongly related with severity of IVDD grades.^8^ Meanwhile, accumulating evidence demonstrated that LPS treatment in cells activated TLR4/NF‐κB signalling axis, which dominates the downstream inflammatory‐related and catabolic‐related genes expression.^13^, ^29^ Therefore, we used LPS to mimic IVDD progression. And our cell experiment results showed that LPS activated TLR4/MyD88 and NF‐κB signalling, increased secretion and generation of ADAMTS‐5, MMP‐13, TNF‐a, iNOS, IL‐6 and COX‐2, accelerating ECM breakdown in NP cells. However, Pro‐B3 pre‐treatment reversed these phenomena.

TLR4 is a conserved transmembrane protein as a member of toll‐like receptors family dominating multiple downstream inflammatory pathways in mammal.^30^ Actually, TLR4 associates with MD‐2 thereby forming TLR4–MD‐2 heterodimer, which recognizes and interacts with pathogen‐related molecular patterns (PAMPs) such as LPS.^31^ In brief, LPS binding medicated the creation of an m‐shaped receptor multimer, comprising two copies of the TLR4–MD‐2–LPS complex arranged symmetrically.^9^ Five of the six lipid chains of LPS bind with a large hydrophobic pocket in MD‐2 and the remaining chain is exposed to the surface of MD‐2, which forms a hydrophobic bond with the conserved phenylalanines of TLR4.^9^ Our ELISA and co‐immunoprecipitation results showed that Pro‐B3 inhibited TLR4/MD‐2/LPS complex formation by intervening the binding of LPS to MD‐2, and docking analysis also indicated Pro‐B3 molecule specifically linked with the GLU‐92 of MD‐2 by forming a hydrogen bond. In the human monocyte cell line, treatment with Pro‐B1 down‐regulated LPS‐activated TLR4, p38 MAPK and NF‐κB signalling pathways, which are partially consistent with our results.^32^ Interestingly, Sung et al reported that Pro‐B2 obviously suppressed LPS‐induced inflammatory responses but negatively affect TLR4 signalling in macrophages.^33^

Next, TLR4‐MD‐2‐LPS complex formation leads to juxtaposition of the intracellular domains of TLR4, which is also termed as Toll/IL‐1R homology (TIR) domains.^34^, ^35^ TIR domain of TLR4 binds with TIR domain of MyD88, subsequently links with another TIR containing adaptor protein, referred to as MyD88 adaptorlike (Mal). In addition to TIR domain, MyD88 possesses a death domain, which interacts with the death domain of IRAK to activate TRAF6‐IKKs‐NF‐κB axis signalling.^36^ The level of MyD88, TLR4 and TRAF6 was increased and NF‐κB signalling activation in IVDD tissue, compare to the normal NP tissue.^8^, ^12^ Similarly, our cell experiments found that treatment NP cells with LPS up‐regulated TRAF6, MyD88, and IRAK1 expression and stimulated NF‐κB signalling, while Pro‐B3 alleviates these changes.

NF‐κB pathway has an essential role in IVDD pathogenesis.^37^, ^38^ In the context inflammation caused by LPS, IκBα becomes phosphorylated, which triggers the translocation of p65. Several researches have shown that p65 promotes transcription of inflammatory genes, for example PGE2, IL‐6, COX‐2, iNOS, and TNF‐α. NO stimulates MMPs generation which then inhibits ECM production.^39^ PGE2 is derived from COX‐2 and is involved in cartilage breakdown by ADAMTS‐5 and MMPs.^40^ MMP‐13 is a collagenase with the high specificity for collagen II breakdown and ADAMTS‐5 expedites cytokine‐triggered aggrecan degradation.^41^ This study display that Pro‐B3 can significantly inhibit overproduction of NO, PGE2, iNOS, and COX‐2 proteins in NP cells treated with LPS. The same effects were observed for TNF‐α and IL‐6. Asou et al reported that Pro‐B3 also exhibits anti‐inflammatory effect in IL‐1β‐exposed chondrocytes16. For NF‐κB pathway, Pro‐B3 attenuated LPS‐mediated nuclear transfer of p65. Besides, other individual compound of proanthocyanidins showed the inhibition of NF‐κB signalling such as Pro‐B1, Pro‐B2 and Pro‐C1 in various cell model.^18^

For in vivo experiment, we established rat tail vertebra puncture model to evaluate the protection of Pro‐B3 for IVDD. The rat in IVDD group showed less gelatin NP cells, obvious fibrochondrocytes and structural failure of AF, accompanied with histological score. However, Pro‐B3 ameliorated these phenomena, reducing the histological score in IVDD mice.

In summary, we demonstrated that Pro‐B3 inhibit LPS‐stimulated inflammatory reaction and ECM breakdown by blocking the TLR4‐NF‐κB signalling axis. In addition, oral administration of Pro‐B3 abolished IVDD progression in surgery‐induced IVDD rat model. The data of current study indicated the potential of Pro‐B3 in the prevention and treatment of IVDD.

## CONFLICT OF INTEREST

The authors declare no conflict of interest.

## AUTHOR CONTRIBUTIONS

PS and ZCH performed experiments and wrote the manuscript; QT answered the reviewer's questions and revised the manuscript; SYH, YZH and JHZ analysed data to form graphs; YZH, JHZ and HXL designed the experiments; HXL helped to write and modificate the manuscript.

## Data Availability

The data used to support the findings of this study are available from the corresponding author upon request.
