# Supplementary material for: Procyanidin B3 alleviates intervertebral disc degeneration via interaction with the TLR4/MD‐2 complex
Source: J Cell Mol Med. 2020 Feb 18;24(6):3701–11. doi: 10.1111/jcmm.15074 (PMC7131944; doi:10.1111/jcmm.15074)
Supplement: Supplementary file 1 [file JCMM-24-3701-s001.docx]

**Histological Grading Scale[**[**1**](#_ENREF_1)**]**

***I. Cellularity of the anulus fibrosus***

Grade:

1. Fibroblasts comprise more than 75% of the cells

2. Neither fibroblasts nor chondrocytes comprise more than 75% of the cells

3. Chondrocytes comprise more than 75% of the cells

***II. Morphology of the anulus fibrosus***

Grade:

1. Well-organized collagen lamellae without ruptured or serpentine fibers

2. Inward bulging, ruptured or serpentine fibers in less than one third of the annulus

3. Inward bulging, ruptured or serpentine fibers in more than one third of the annulus

***III. Border between the anulus fibrosus and nucleus pulposus***

Grade:

1. Normal, without any interruption

2. Minimal interruption

3. Moderate or severe interruption

***IV. Cellularity of the nucleus pulposus***

Grade:

1. Normal cellularity with stellar shaped nuclear cells evenly distributed throughout the nucleus

2. Slight decrease in the no. of cells with some clustering

3. Moderate or severe decrease (>50%) in the number of cells with all the remaining cells clustered and separated by dense areas of proteoglycans

***V. Morphology of the nucleus pulposus***

Grade:

1. Round, comprising at least half of the disc area in midsagittal sections

2. Rounded or irregularly shaped, comprising one quarter to half of the disc area in midsagittal sections

3. Irregularly shaped, comprising less than one quarter of the disc area in midsagittal sections

The scale is based on 5 categories of degenerative changes with scores ranging from 5 points (1 in each category) for a normal disc to 15 points (3 in each category) for a severely degenerated disc.

1. **Han B, Zhu K, Li F-c, Xiao Y-x, Feng J, Shi Z-l, Lin M, Wang J, Chen Q-x.** A Simple Disc Degeneration Model Induced by Percutaneous Needle Puncture in the Rat Tail. *Spine*. 33: 1925-34.
